# Case Report: A Boy From a Consanguineous Family Diagnosed With Congenital Muscular Dystrophy Caused by Integrin Alpha 7 (*ITGA7*) Mutation

**DOI:** 10.3389/fgene.2021.706823

**Published:** 2021-09-06

**Authors:** Wenqing Xia, Zhumei Ni, Zheng Zhang, Hongfei Sang, Huifang Liu, Zhenzhen Chen, Lin Jiang, Congguo Yin, Jinyu Huang, Lingfei Li, Xiaoguang Lei

**Affiliations:** ^1^The Fourth School of Clinical Medicine, Zhejiang Chinese Medical University, Hangzhou, China; ^2^Department of Neurology, Hangzhou First People's Hospital, Hangzhou, China; ^3^Division of Neurology, Department of Medicine, University of Hong Kong, Hong Kong, SAR China; ^4^Department of Hematology, Affiliated Hangzhou First People's Hospital, Zhejiang University, Hangzhou, China; ^5^Department of Cardiology, Affiliated Hangzhou First People's Hospital, Zhejiang University School of Medicine, Hangzhou, China; ^6^Department of Neurology, First Affiliated Hospital of Kunming Medical University, Kunming Medical University, Kunming, China

**Keywords:** congenital muscular dystrophy, a consanguineous family, rare genetic mutation, genetic consultation, integrin alpha 7

## Abstract

**Introduction:** Congenital muscular dystrophy (CMD) is a group of early-onset disorders with clinical and genetic heterogeneity. Patients always present with muscle weakness typically from birth to early infancy, delay or arrest of gross motor development, and joint and/or spinal rigidity. There are various genes related to the development of CMD. Among them, mutations in integrin alpha 7 (*ITGA7*) is a rare subtype. The identification of disease-causing genes facilitates the diagnosis and treatment of CMD.

**Methods:** We screened *ITGA7* mutations in four people by whole exome sequencing and targeted sequencing from a consanguineous family. We then carried out electromyography and neuroelectrophysiological examinations to clarify a clinical picture of the patient diagnosed with CMD.

**Results:** We report a Chinese boy diagnosed with CMD who carries a homozygous variant (c.1088dupG, p.H364Sfs^*^15) of the *ITGA7* gene. According to the genotype analysis of his family members, this is an autosomal recessive inheritance.

**Conclusions:** Our case further shows that *ITGA7* mutation is related to CMD. Genetic counseling and multidisciplinary management of CMD play an important role in helping patients and their family. Further elucidation of the significant clinical and genetic heterogeneity, therapeutic targets, and the clinical care for patients remains our challenge for the future.

## Introduction

Congenital muscular dystrophy (CMD) is a group of hereditary muscular dystrophy beginning at birth or early life. The clinical manifestations are postnatal hypotonia and motor development, joint contracture, and increased serum creatine kinase (CK) (Bonne et al., 2018). Initially, CMD has been diagnosed based on clinical features and histopathology. Nowadays, CMDs are classified into different subtypes according to the pathogenetic genes (Hayashi et al., 1998). According to the 2019 version of the GeneTable, there are 34 genes related to CMD phenotypes (Bonne et al., 2018). Among them, laminin subunit alpha 2–related (merosin deficient), collagen VI–related, and α-dystroglycan–related CMDs are the most common types. The underlying mechanisms are predominantly related to disruption of components of the muscle extracellular matrix and its interaction with the sarcolemmal membrane. CMD caused by mutation in integrin α7 (*ITGA7*) is a rare subtype. Only a few patients diagnosed with CMD were found to have *ITGA7* mutation (Hayashi et al., 1998; Esposito et al., 2013; Yu et al., 2017; Karaca et al., 2018). Here, we report a rare case of a boy from a consanguineous family diagnosed with CMD caused by *ITGA7* mutation.

## Case Description

**Figure 1:**
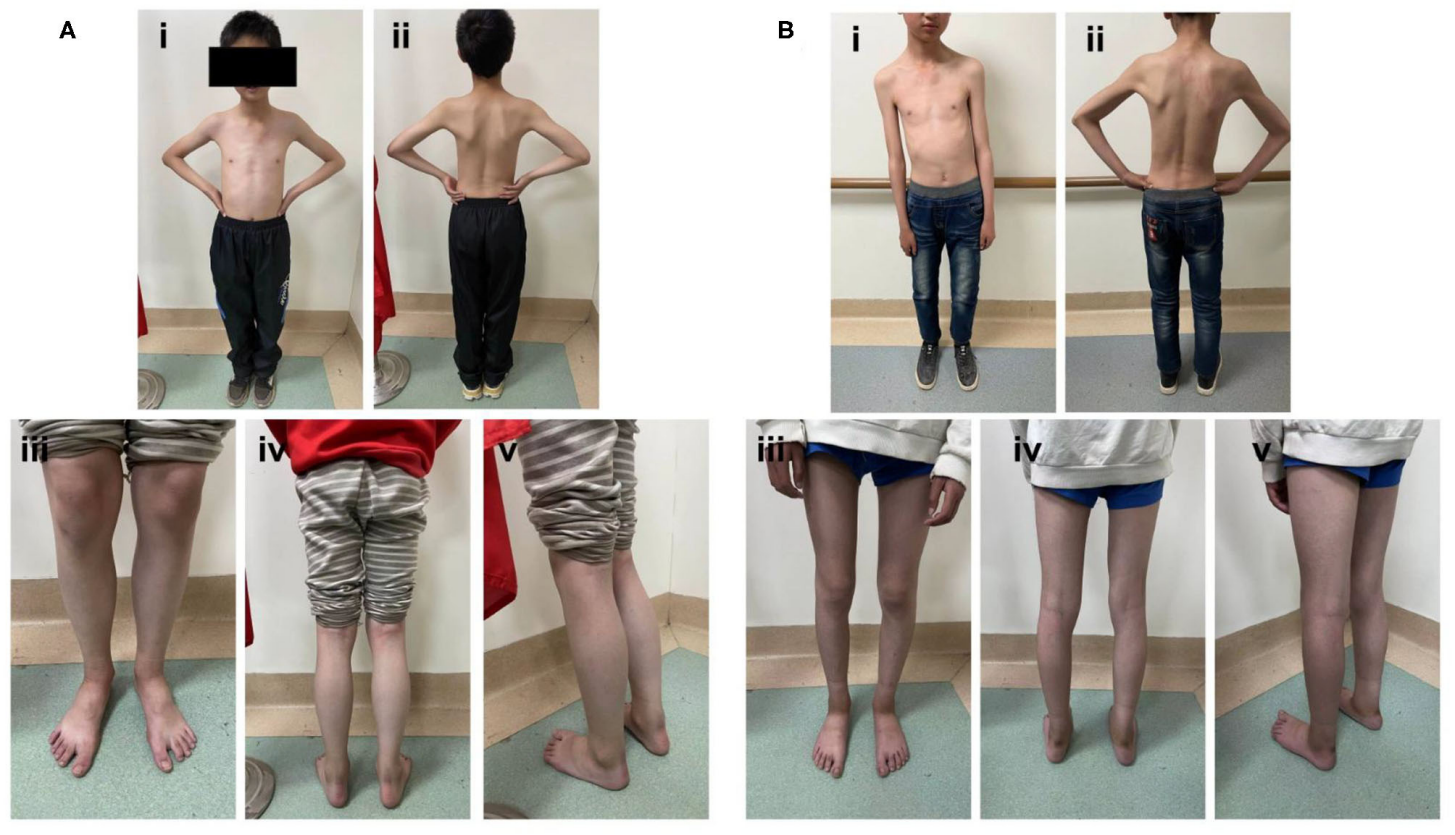
Photographs of the proband at 10 years old. **(A, B)** The proband presented with obvious limb muscular atrophy and waddling gait. A neurological examination showed proximal muscle weakness.

The proband is the second child in the family, aged 10 years old. The boy presented with muscle weakness at the age of 3 years old and the symptoms were slowly progressive. He had difficulties in squatting in daily life and sit-ups with knee flexed in excise. He walked on tiptoes. Physical examination showed proximal muscular atrophy of both limbs (Figure 1A). The patient walked in a gait indicating proximal muscular atrophy. Lab investigation showed significantly increased serum creatine kinase (CK) activity (286 U/L, normal = 24–195 U/L). We also found increased red blood cell count (6.22 × 10^12^/L, normal range: 3.5–5.5 × 10^12^/L), decreased mean cell volume (MCV, 65.60 fl, normal range: 82–95 fl) and mean cell hemoglobin (MVH, 21.20 pg, normal range: 27–31 pg), and increased platelets (PLT). The serum electrolytes, calcium, lactate, and thyroid function test results were normal. EMG showed multiple lesions and most muscles showed neurogenic lesion, especially in both quadriceps femoris. There was muscular lesion in part of the tibialis anterior muscle. There was no abnormality in the cardiovascular or respiratory system. Echocardiography was normal. Muscle biopsy was not performed due to parental preference. Whole exome sequencing showed that the patient has a homozygous variant (c.1088dupG, p.H364Sfs^*^15) of the *ITGA7* gene.

**Figure 2:**
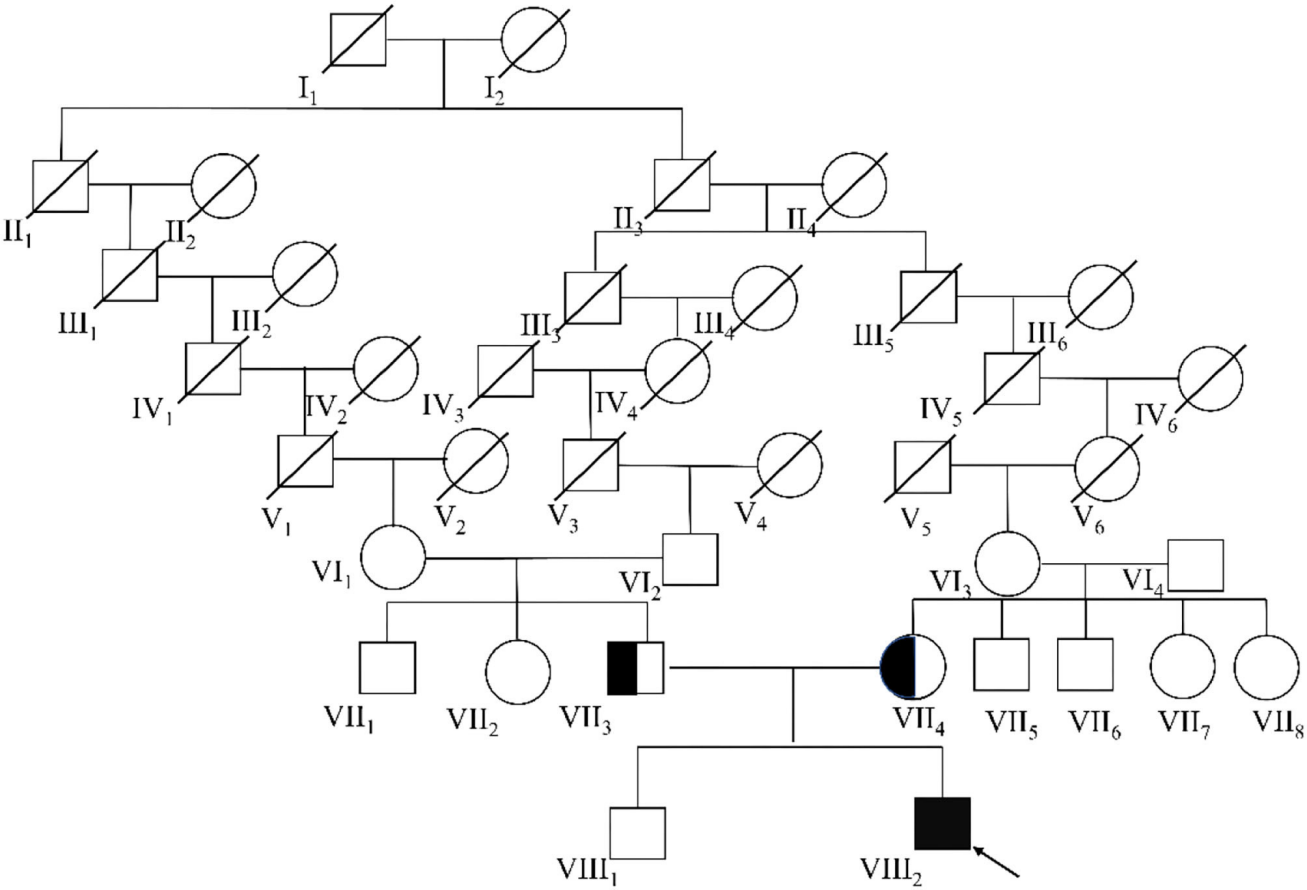
The complete eight-generational Chinese pedigree. The proband affected by *ITGA7* is indicated with an arrow and a dark box.

**Figure 3:**
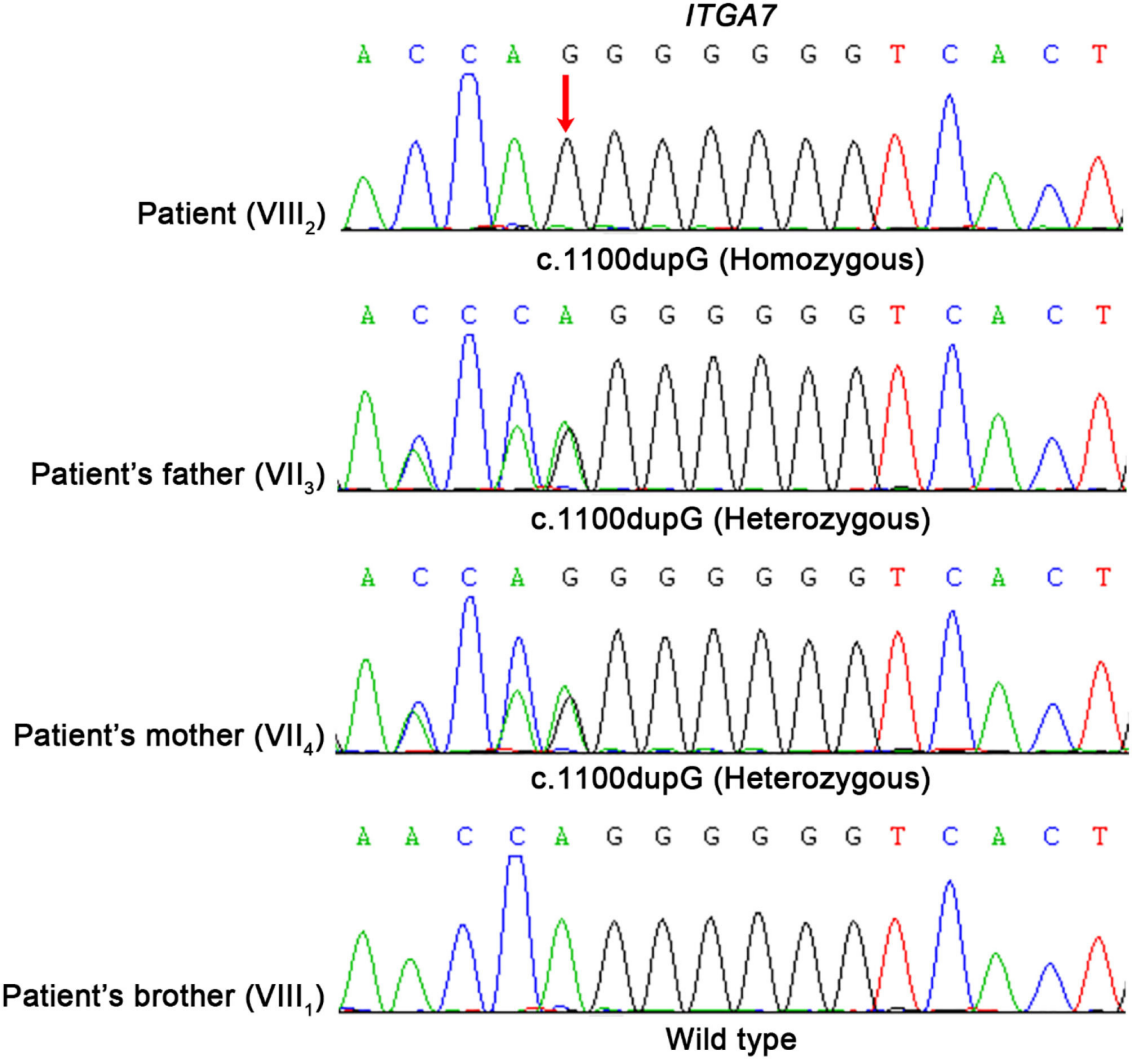
Molecular analysis of *ITGA7* gene mutation. Mutation analysis of the *ITGA7* gene. Red arrowhead marks the sites of base alterations. The DNA sequences of the family subjects are shown; the pedigree number is indicated on the left.

The big brother of the proband presented with recurrent pain of the lower limbs, mild muscle wasting, and weakness of the upper limbs. He was thin and unable to unscrew a bottle cap. He had no difficulty in walking and running. Physical examination found obvious scoliosis and atrophy of the limb muscle (Figure 1B). Lab investigation showed increased red blood cell count (6.42 × 10^12^/L, normal range: 4.2–5.5 × 10^12^/L), decreased MCV and MVH, and increased PLT. Slightly increased angiotensin-converting enzyme (ACE, 89.5 U/L, normal range: 5.0–89.0 U/L) marked increased alkaline phosphatase (ALP, 324.1 IU/L, normal range: 45.0–125.0 IU/L), normal CK (102.0 IU/L, normal range: 50.0–310.0 IU/L), and slightly increased creatine phosphokinase myocardial band (CK-MB, 24.1 U/L, normal range: <24.0 U/L). NCV showed that the brother has left quadriceps muscular disruption. Others (MRI of thoracic vertebra, lower limbs) are normal. The symptoms were relieved after vitamin B12 treatment. Two kids are being followed up at a pediatric tertiary care hospital with physiotherapy and rehabilitative care. Their parents had no symptoms and Sanger sequencing confirmed that they have heterozygous mutation in *ITGA7* (Figures 2, 3).

## Methods

### Clinical Assessment

The clinical assessment of the patient comprised a neurological behavior examination and evaluation of his nerve conduction velocity is detailed in Supplementary Tables 1, 2.

### Whole Exome Sequencing

Peripheral blood (5 ml) of each child and their parents was collected. DNA was extracted using whole blood magnetic bead purification kit. Full exon genes were captured by IDT_X_Gen Exome Research Panel v1.0 and sequenced on Illumina Novaseq 6000 platform. Afterwards, the sequencing data were evaluated according to Illumina Sequence Control Software (SCS) and analyzed as follows. The original sequencing data were deconstructed, removing the joint sequences, and then filtered and aligned to the NCBI database of human reference genome (hg19) using the BWA software (http://bio-bwa.sourceforge.net/). The single-nucleotide mutation [single-nucleotide variation (SNV)] and absence of insertion mutation (inserts and deletions, INDEL) were called using GATK software (https://software.broadinstitute.org/gatk/) and annotated by ANNOVAR software (http://annovar.openbioinformatics.org/en/latest/). All candidate variants were filtered first against 1,000 genomes project database, for a minor allele frequency (MAF) ≥ 1%, and ExAC hom AC ≥ 3. Obtained variants were further selected according to co-segregation, genetic model, and a MAF <1% in three databases (1,000 genomes project_EAS, ExAC, and gnomAD_EAS). After analyzing the sequencing results as above, a variant of *ITGA7* was found in the child, which had not been reported in the HGMDPRO database.

### Sanger Sequencing

The candidate causal genes discovered *via* WES were then confirmed by Sanger sequencing and co-segregation analyses among the family were also conducted. The primers were designed using Primer Premier 5.0 (Premier Biosoft, USA) and PCR was carried out to amplify the fragments covering the mutated sites on LifeECO Thermal Cycler TC-96/G/H(b)C (Bioer Technology Co. Ltd., China). The PCR products were further purified with agarose gel electrophoresis and then sequenced by ABI 3730XL DNA Sequencer (Applied Biosystems, Thermo Fisher Scientific, USA). Sanger sequencing results were analyzed by Chromas Lite v2.01 (Technelysium Pty Ltd., Tewantin, QLD, Australia).

## Discussion and Conclusion

To date, only a few *ITGA7* variants have been reported, and it is not clear whether all variants are pathogenic mutations (Table 1). In 1998, Hayashi et al. first described three patients with CMD, two of whom carried the ITGA7 mutation and one did not. The first patient had one 21-bp insertion mutation on one allele and a 98-bp deletion on the other allele of the *ITGA7* gene, which were both splice mutations. The second patient had the same 98-bp deletion and had a 1-bp frame-shift deletion in the other allele, which is a compound heterozygote. The third patient showed a marked deficiency in the *ITGA7* mRNA, but no mutations in the coding region were described (Hayashi et al., 1998). The three patients reported were affected with congenital myopathy with variable clinical phenotype, and all showed a complete absence of integrin α7 in their muscle biopsies due to primary integrin α7 nonsense/splicing mutations or to a downregulation of integrin α7 mRNA (Hayashi et al., 1998). In 2013, Esposito et al. reported a female with homozygous missense mutations in two genes, the myosin heavy chain 7B and the *ITGA7*, who presented with complicated congenital myopathy with left ventricular non-compact cardiomyopathy (Esposito et al., 2013). Later in 2017, Yu et al. described a female with proximal muscle weakness, who had c.2701A>G and c.1828G>A in *ITGA7* (Yu et al., 2017). In 2018, Karaca et al. found an individual with a deleterious variant in *ITGA7*, conferring a molecular diagnosis of CMD. This proband has microcephaly, agenesis of the corpus callosum, cerebellar hypoplasia, seizures, horseshoe kidney, scoliosis, hemivertebrae, asymmetric extremities, and hypopigmented skin macules (Karaca et al., 2018). Although the case described above had various symptoms and different grades of severity, the consistent clinical features were muscle weakness and increased CK level, which were also found in our proband. Among the published studies, patients reported by Esposito's team had combined mutations (Esposito et al., 2013). This condition was not found in our patient.

**Table 1:** Characteristics of CMD induced by *ITGA7* mutation (NM_002206.3).

**References**	**Gender**	**Age at onset**	**Parental consanguinity**	**Family history**	**Clinical features**	**Investigation findings**	**Genetic variation**
Hayashi et al. (1998)	M	15 months	No	No	• Could not jump or run • Mental retardation • Delayed motor milestones • Verbal abilities: limited to only a few words	• Normal brain MRI and EEG • CK: 528 IU/L • Muscle biopsy: mild fiber size variation and mild type 1 fiber predominance (65%) with no evidence of myofiber necrosis or regeneration	c.1506-2A>G c.2712+2T>C
	F	2 months	No	No	• Could not run and climb stairs without support • Congenital dislocation of hip and torticollis • Gower's sign and wadding gait • No cognitive impairment	• CK: 236 IU/L • Muscle biopsy: substantial fatty replacement and fiber size variation	c.2712+2T>C c.1205delG (p.Gly402Valfs*104)
	M	8 months	No	No	• Hypotonia and torticollis • Delayed motor milestones • No mental retardation	• CK:163 IU/L • Muscle biopsy: mild fiber size variation	No mutation, (only marked deficiency of *ITGA7* mRNA)
Esposito et al. (2013)	F	At birth	NA		• Poor sucking, hypotonia, persistent crying, difficulties in chewing and swallowing • Tendon reflexes were absent • Scoliosis	• CK: normal • Muscle biopsy: predominance of type 1 fibers (76%), the mean diameter of which was 30% smaller than that of the type 2 fibers, dystrophin normal	c.2644G>A (p.E882K)
Yu et al. (2017)	F	2 years old	NA		• Proximal muscle weakness with facial weakness and dropping head • Contracture	• CK:160 IU/L • Pathological findings: dystrophic • Grade of the weakest muscle group: severe	c.2701A>G (p.I901V) c.1828G>A (p.G610R)
Karaca et al. (2018)	Family	NA	NA	Yes	**-Proband:** seizures, scoliosis, asymmetric extremities **-Other family members:** hypotonia, scoliosis, microcephaly, agenesis of ^C^CC, cerebellar hypoplasia, seizure, chorioretinal lacunae, asymmetric extremities, hypopigmented macules, horseshoe kidney, and hemivertebrae	NA	NA
Xia et al. (2021)	M	10	Yes	No	• Muscle weakness: difficulties in squatting in daily life and sit-ups with knee flexed in excise	• ALP: 324.1IU/L • CK: normal	c.1088dupG p.H364Sfs*15

*M, male; F, female; NA, not available*.

Integrins are a group of heterodimeric integral membrane glycoproteins with diverse combinations of α and β subunits. They mediate cell-to-cell and cell-to-matrix interactions, thus playing roles in cell migration, morphologic development, differentiation, and metastasis (Di Maggio et al., 2017). *ITGA7* (GenBank: NG_012343.1) is on chromosome 12. This gene has 28 exons, among which 26 code for protein. *ITGA7* has been reported to be associated with various types of cancers (Guan et al., 2020), stem cell differentiation (Zhang et al., 2019), and skeletal muscle development (Rooney et al., 2012; McClure et al., 2016). Mice lacking integrin α7 have demonstrated impaired muscle healing after cardiotoxin injury (Rooney et al., 2012). Previous studies indicate that the integrin α7 subunit is upregulated during myoblast differentiation. Myoblasts with silenced integrin α7 were found to regulate myogenic differentiation and demonstrate defective fusion (McClure et al., 2016). Loss of integrin α7 exacerbates a newly discovered muscle phenotype in mice lacking major adhesion complexes in skeletal muscle (Marshall et al., 2012). *ITGA7*-expressing muscle-resident glial cells can be activated by loss of neuromuscular junction integrity (Proietti et al., 2021). *ITGA7*-expressing muscle-resident glial cells are activated by loss of neuromuscular junction integrity, indicating α7 in late differentiation (Proietti et al., 2021). The homozygous mutation found in our study led to a shift in the reading frame, resulting in truncating proteins with 15 miscoded amino acids (Supplementary Figure 1). The expression of biologically functional proteins was absent, causing CMD in our patient as a result. However, we did not obtain muscle biopsy, so we cannot prove that the patient lacks the ITGA7 protein.

Despite the recent advances in our understanding of the molecular basis of neuromuscular disorders, the underlying molecular defect can be identified only in a subset of the cases. CMD present with a wide spectrum of severity and onset. Progression and other features are variable depending on the subtype and severity of the specific genetic mutation.

## Data Availability Statement

The datasets for this article are not publicly available due to concerns regarding participant/patient anonymity. Requests to access the datasets should be directed to the corresponding author.

## Ethics Statement

The studies involving human participants were reviewed and approved by Review Board of the Affiliated Hangzhou First People's Hospital, Zhejiang University School of Medicine. Written informed consent to participate in this study was provided by the participants' legal guardian/next of kin. Written informed consent was obtained from the individual(s), and minor(s)' legal guardian/next of kin, for the publication of any potentially identifiable images or data included in this article.

## Author Contributions

WX, ZN, and LL designed the study. ZZ, HS, and ZC performed the genetic analysis and bioinformatics evaluations. LL and XL drafted the manuscript. LJ, CY, and JH conducted the clinical evaluations. All authors analyzed the data and approved the final manuscript.

## Conflict of Interest

The authors declare that the research was conducted in the absence of any commercial or financial relationships that could be construed as a potential conflict of interest.

## Publisher's Note

All claims expressed in this article are solely those of the authors and do not necessarily represent those of their affiliated organizations, or those of the publisher, the editors and the reviewers. Any product that may be evaluated in this article, or claim that may be made by its manufacturer, is not guaranteed or endorsed by the publisher.

## Supplementary Material

Supplementary Figure 1 Schema of integrin alpha-7 with the mutation found in our patient. This homozygous mutation leads to a shift in the reading frame, resulting in truncating proteins with 15 miscoded amino acids.

Supplementary Table 1 Nerve conduction studies of the proband. L, left; R, right; Lat, latency; Amp, amplitude; Dis, distance; CV, conduction velocity.

Supplementary Table 2 Nerve conduction studies of the brother of the proband. L, left; R, right; Lat, latency; Amp, amplitude; Dis, distance; CV, conduction velocity.
