# Supplementary figures and images for: Case Report: A Boy From a Consanguineous Family Diagnosed With Congenital Muscular Dystrophy Caused by Integrin Alpha 7 (ITGA7) Mutation
Source: Front Genet. 2021 Sep 6;12:706823. doi: 10.3389/fgene.2021.706823 (PMC8450528; doi:10.3389/fgene.2021.706823)

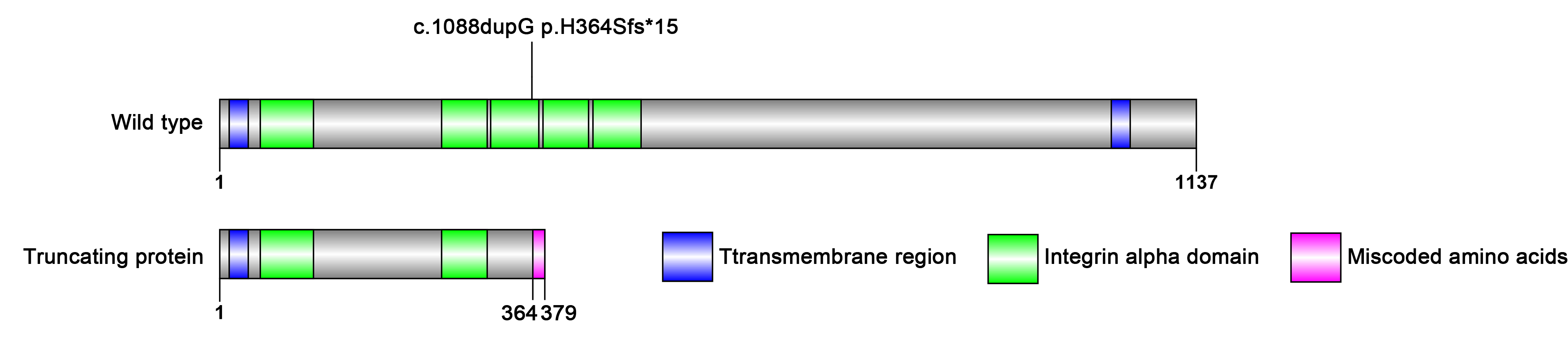

Supplement: Supplementary Figure 1 — Schema of integrin alpha-7 with the mutation found in our patient. This homozygous mutation leads to a shift in the reading frame, resulting in truncating proteins with 15 miscoded amino acids. [file Image_1.jpg]
