# Supplementary material for: Case Report: A Boy From a Consanguineous Family Diagnosed With Congenital Muscular Dystrophy Caused by Integrin Alpha 7 (ITGA7) Mutation
Source: Front Genet. 2021 Sep 6;12:706823. doi: 10.3389/fgene.2021.706823 (PMC8450528; doi:10.3389/fgene.2021.706823)
Supplement: Supplementary Table 1 — Nerve conduction studies of the proband. L, left; R, right; Lat, latency; Amp, amplitude; Dis, distance; CV, conduction velocity. [file Table_1.docx]

**Supplementary Table 1: Nerve conduction studies of the proband**

| **Parameters** | **Motor nerve** | | | | | | | | | **Sensory nerve** | | | |  |
| --- | --- | --- | --- | --- | --- | --- | --- | --- | --- | --- | --- | --- | --- | --- |
|  | Femoralis | | Tibialis | | Peroneus communis | | nervi peronaeus superficialis | | | | Peroneus communis | | | |
|  | L | R | L | R | L | R | L | | R | | L | | R | |
| Lat (ms) | 6.17 | 6.29 | 5.83 | 5.85 | 8.2 | 9.0 | - | | - | | - | | - | |
| Amp (μV) | 10.9 | 17.3 | 8.8 | 8.5 | 2.5 | 2.8 | - | - | | | 17 | 37.5 | | |
| CV (m/s) | 63.5 | 63.9 | 60.4 | 65 | 58.5 | 50.9 | - | - | | | 58.5 | 59 | | |

L: left; R: right; Lat: latency; Amp: amplitude; Dis: distance; CV: conduction velocity
