# Supplementary material for: Case Report: A Boy From a Consanguineous Family Diagnosed With Congenital Muscular Dystrophy Caused by Integrin Alpha 7 (ITGA7) Mutation
Source: Front Genet. 2021 Sep 6;12:706823. doi: 10.3389/fgene.2021.706823 (PMC8450528; doi:10.3389/fgene.2021.706823)
Supplement: Supplementary Table 2 — Nerve conduction studies of the brother of the proband. L, left; R, right; Lat, latency; Amp, amplitude; Dis, distance; CV, conduction velocity. [file Table_2.docx]

**Supplementary Table 2: Nerve conduction studies of the brother of the proband**

| **Parameters** | **Motor nerve** | | | | | | | | | **Sensory nerve** | | | | | |
| --- | --- | --- | --- | --- | --- | --- | --- | --- | --- | --- | --- | --- | --- | --- | --- |
|  | Median | | Ulnar | | Sural | | Median | | | | Ulnar | | Sural | | |
|  | L | R | L | R | L | R | L | | R | | L | R | L | | R |
| Lat (ms) | 6.17 | 6.29 | 5.83 | 5.85 | 9.21 | 8.65 | 1.95 | | 1.88 | | 1.77 | 1.71 | 1.11 | | 0.96 |
| Amp (μV) | 10.9 | 17.3 | 8.8 | 8.5 | 5.9 | 6.7 | 39.6 | 29.7 | | | 16.6 | 18.2 | 23.5 | 25.2 | |
| CV (m/s) | 63.5 | 63.9 | 60.4 | 65 | 54.6 | 57.8 | 61.5 | 58.5 | | | 62.1 | 58.5 | 63.1 | 62.5 | |

L: left; R: right; Lat: latency; Amp: amplitude; Dis: distance; CV: conduction velocity
